# The Need for Sex Hormone Analysis in Addition to Long-Term Follow-Up of Phytosterol Supplementation

**DOI:** 10.5935/abc.20180132

**Published:** 2018-08

**Authors:** Heitor Oliveira Santos

**Affiliations:** Universidade Federal de Uberlândia, Uberlândia, MG - Brazil

**Keywords:** Cardiovascular Diseases/prevention&control, Hypercholesterolemia, Phytosterois, Steroids

**Dear Editor,**

I read with great interest the article entitled “Phytosterols in the Treatment of Hypercholesterolemia and Prevention of Cardiovascular Diseases”, by Cabral and Klein, published in the Brazilian Archives of Cardiology.^1^ The authors discuss phytosterol doses in the treatment of hypercholesterolemia, showing the current consensus of renowned guidelines and approval by global regulatory agencies.

As already mentioned, the main phytosterol mechanism of action occurs through the reduction (30% to 50%) in the intestinal absorption of cholesterol.^1^ The authors, however, make it clear that long-term follow-up is essential to assess the association of phytosterol supplementation with the risk of cardiovascular diseases. Additionally, I emphasize another important investigation: analyses of serum sex hormones during randomized clinical trials based on phytosterol administration.

**Figure 1:**
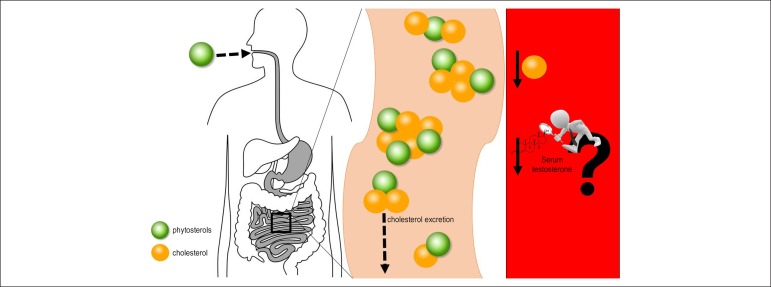
Proposal to analyze serum testosterone levels during phytosterol supplementation. Phytosterols increase the excretion of cholesterol, resulting in serum cholesterol decrease. Since cholesterol is important for the synthesis of sex hormones, the decrease in serum testosterone combined with phytosterol supplementation is a hypothesis. Therefore, the testing of this concept is a useful and, at first, an innovative concept. Dotted arrows indicate the conduction of phytosterols; continuous arrows indicate decreased levels of serum cholesterol and testosterone.

I recently showed that cholesterol intake may be related to increased total testosterone in men, whereas statin use may annul this potential.^2^ Perhaps the use of phytosterols can also attenuate serum total testosterone levels in men (Figure 1).

In one test, the ingestion of 8.6 g/d of phytosterols reflected in the daily excretion of 28 mg cholesterol/g of fecal dry weight, resulting in an increase of 20 mg / g in comparison to the period prior to the test.^3^ These proportions reflect in the daily excretion of approximately 230 mg of fecal cholesterol without the ingestion of phytosterols and 810 mg with the ingestion of phytosterols, since the average daily fecal excretion in humans is 128 g of wet weight, which corresponds to 29 g of dry weight.^4^

I point out that daily intake of 500 to 1,000 mg of cholesterol may result in an approximate increase of 130 ng/dL of total testosterone in men. In rats, the ingestion of phytosterols for 22 days reduced serum testosterone by 33%, in comparison to controls.^5^ To the best of my knowledge, there are no studies that analyzed sex hormones in association with phytosterol administration in humans. Therefore, in addition to the aforementioned need to analyze the long-term administration of phytosterols, it is also important to consider sex hormone measurements in this context.
