# Supplementary material for: The Need for Sex Hormone Analysis in Addition to Long-Term Follow-Up of Phytosterol Supplementation
Source: Arq Bras Cardiol. 2018 Aug;111(2):228–9. doi: 10.5935/abc.20180132 (PMC6122900; doi:10.5935/abc.20180132)
Supplement: Related texts

# Reply

**Authors:** Carlos Eduardo Cabral, Márcia Regina Simas Torres Klein

We thank you for your interest and comments related to our recent review article entitled “Phytosterols in the Treatment of Hypercholesterolemia and Prevention of Cardiovascular Diseases,”^1^ which aimed specifically to address the available evidence in the literature on the association between phytosterols and risk of cardiovascular diseases.

We agree that it is important to investigate the effects of phytosterols on serum levels of sex hormones, especially due to the potential risk of reducing plasma testosterone levels. We are not aware of studies carried out in humans that have performed such an evaluation. Experimental animal studies are scarce, and a reduction in plasma testosterone levels has been observed in some,^2^^,^^3^ but not all of them.^4^

**Sincerely,**

Carlos Eduardo Cabral Márcia Regina Simas Torres Klein

## References

[1] Cabral CE, Klein MR (2017). Phytosterols in the Treatment of Hypercholesterolemia and
Prevention of Cardiovascular Diseases. Arq Bras Cardiol.

[2] Qasimi MI, Nagaoka K, Watanabe G (2017). The effects of phytosterols on the sexual behavior and
reproductive function in the Japanese quail (Coturnix coturnix
japonica). Poult Sci.

[3] Awad AB, SriHartati M, Fink CS (1998). Phytosterol feeding induces alteration in testosterone
metabolism in rat tissues. J Nutr Biochem.

[4] Ryökkynen A, Käyhkö UR, Mustonen AM, Kukkonen JV, Nieminen P (2005). Multigenerational exposure to phytosterols in the
mouse. Reprod Toxicol.
